# Predicting severe COVID-19 using readily available admission indicators: SpO2/FiO2 ratio, comorbidity index, and gender

**DOI:** 10.3389/ebm.2024.10193

**Published:** 2024-11-20

**Authors:** Luan D. Vu, Rebecca C. Christofferson, Hollis R. O’Neal, Diana Hamer, Anh T. Q. Phan, Katie M. Vance, E. A. Turner, Avinash Kumar, Ibrahim Musa Yola, Natalie Lim, Beverly Ogden, Stephania A. Cormier

**Affiliations:** ^1^ Department of Biological Sciences, Louisiana State University and Pennington Biomedical Research Center, Baton Rouge, LA, United States; ^2^ Department of Pathobiological Sciences, School of Veterinary Medicine, Louisiana State University, Baton Rouge, LA, United States; ^3^ Medical Director of Research, Our Lady of the Lake Regional Medical Center, Pulmonary and Critical Care Medicine, Louisiana State University Health Sciences Center, Baton Rouge, LA, United States; ^4^ Office of Research Administration, Our Lady of the Lake Regional Medical Center, Baton Rouge, LA, United States; ^5^ Department of Research, Woman’s Hospital, Baton Rouge, LA, United States

**Keywords:** COVID-19, SARS-CoV-2, prognostic, Charlson Comorbidity Index, severity

## Abstract

The focus of this study was to identify risk factors for severe and critical COVID-19, evaluate local respiratory immune responses to SARS-CoV-2 infection, and develop a prognostic tool for COVID-19 severity using accessible early indicators. Using nasopharyngeal swab samples from hospitalized patients with COVID-19 of varying severity during the first wave of the pandemic from March to May 2020 in Louisiana, we evaluated the association between COVID-19 severity and viral load, respiratory immune mediators, and demographic/clinical factors. We found that the SpO_2_/FiO_2_ ratio at triage, total comorbidity burden (represented by Charlson Comorbidity Index), and gender were significantly associated with COVID-19 severity. Using these early significant indicators, we developed a prognostic tool for COVID-19 severity that is simple and convenient. Additionally, our study demonstrated that elevated levels of respiratory immune mediators, including IL-10, IL-6, MCP-1, and MCP-3, were significantly associated with COVID-19 severity. We also found that viral load at the time of admission was associated with disease severity. Our findings highlight the feasibility and importance of evaluating the humoral component of local mucosal immune responses and viral load at the infected site using convenient nasopharyngeal swab samples, which could be an effective method to understand the relationship between viral infection and immune responses at the early stages of infection. Our proposed prognostic tool has the potential to be useful for COVID-19 management in clinical settings, as it utilizes accessible and easy-to-collect variables at the time of admission.

## Impact statement

Early identification of COVID-19 severity indicators is vital for managing severe cases, allowing timely interventions to minimize complications and fatalities. Our study devised a practical prognostic tool for clinical settings, utilizing easily accessible admission variables like oxygen saturation, Charlson Comorbidity Index, and gender. Furthermore, we highlighted the feasibility of evaluating the humoral component of local mucosal immune responses and viral load using routine nasopharyngeal swab samples. This approach offers valuable insights into infection onset and informs targeted interventions, ultimately reducing COVID-19 related complications and mortality.

## Introduction

The virus that causes the COVID-19 disease, SARS-CoV-2, has caused significant morbidity and mortality worldwide, with over 776 million cases and 7 million deaths attributed to the virus globally as of September 2024 [1]. The initial outbreak in Louisiana, which began in March 2020, caused a tremendous strain on the healthcare system [2, 3], leading to efforts to identify early indicators of COVID-19 severity to manage critical cases effectively. Rapid deterioration of respiratory function, dysregulated host response, and subsequent multiple organ failures are hallmarks of severe COVID-19 and are associated with a high mortality rate [4]. The treatment of severe COVID-19 continues to be challenging and arduous. Timely intervention based on early indicators of COVID-19 severity is essential to reduce mortality and COVID-19-related complications [5, 6].

During the first pandemic wave (from 24th February to 31st July, 2020), certain co-morbidities were indicated as primary risk factors for hospitalization and severe disease outcomes, including diabetes, obesity, COPD/smoking, and chronic kidney disease [7, 8], with additional demographic risk factors based on age and race [7, 9].

Since the emergence of the COVID-19 pandemic, understanding the relationship between viral load and disease severity has also been a critical research topic. Several studies have investigated this association, and the results have been mixed. It was suggested that viral load, often proxied by the qRT-PCR cycle threshold (CT or CQ value), is correlated with disease severity and/or presentation across several studies [10–19]. For example, one study found that higher viral load in patients was correlated with a loss of smell/taste, though there was no significant difference in the presentation of other symptoms [20]. Other studies demonstrated a higher load in severe vs. mild patients [14, 21, 22], or with the risk of death [23–27].

However, the association between viral load and disease severity or symptoms was not observed in other studies of COVID-19, as several studies reported no difference in viral load between asymptomatic patients and symptomatic patients [28–32], or between severity of disease, gender, race identity, or age groups [33–35]. One study found higher viral loads in non-hospitalized patients [36]. Thus, no clear or consistent association between viral load and disease state has emerged.

In addition to co-morbidities and viral loads, lymphopenia elevated inflammatory markers in peripheral blood have been consistently identified as biomarkers for COVID-19 severity. Studies have shown that greater levels of NLRP3 inflammasome activation in peripheral blood corresponded with more severe COVID-19 [37], that low expression of the IFNAR2 gene (an IFN 1 receptor subunit) was associated with critical illness in COVID-19 patients [38], that loss-of-function mutations in an another IFN I receptor subunit (IFNAR1) was associated with severe COVID-19 cases [39], and that auto-antibodies to IFN I were identified as a potential factor for severe COVID-19 [40]. It is evident that increased circulating IL-10, IL-6, IFN-gamma-inducible protein 10 (IP-10), and monocyte chemoattractant protein-1 and -3 (MCP-1 and MCP-3) are significantly associated with COVID-19 severity [41–47]. However, these circulating biomarkers are often observed during the late acute phase of the disease and usually result from the disease severity [44–47]. The evaluation of the immune responses in the peripheral blood compartment during the later phase of the disease may not accurately reflect the early responses of local mucosal immunity – the upper respiratory tract, which is essential for the first-line defense against SARS-CoV-2 and shaping adaptive immune responses. There is a need to better characterize the local innate immune responses to SARS-CoV-2 during the early stage of the disease. At the beginning of this first wave in March 2020, we pivoted our academic research lab into a CLIA-approved testing facility and partnered with local and state-wide facilities to provide enhanced testing capabilities [2]. During this time, we received nasopharyngeal swab samples from hospitalized patients with a full spectrum of COVID-19 severity (from mild, moderate to severe, and critical), spanning the first wave of the pandemic. Such cohorts allowed us to investigate the tripartite problem of how individual risk factors and co-morbidities, early local immune responses to SARS-CoV-2 infection, and relative viral load are associated with disease presentation and outcomes during the first wave of hospitalized patients as this represents the emergence phenomenon.

## Materials and methods

### Study approval

Our retrospective study was conducted at River Road Testing Lab [2], Louisiana State University (LSU), Pennington Biomedical Research Center, and Our Lady of the Lake Regional Medical Center. The study protocol was reviewed and approved by LSU Health Sciences Center [IRB#20-979 and exempt under 45CFR46.104 (d), category 4] on May 14, 2020. The protocol was conducted in accordance with relevant guidelines and institution policies. Because remnant nasopharyngeal swab samples received during routine care from SARS-CoV-2 infected patients were utilized in our study, informed consent was waived. All study sites worked under their approved biosafety protocols for handling SARS-CoV-2 specimens. Additionally, the authors vouch for the accuracy of the data reported.

### Study participants

Because age is a significant predictor for COVID-19 severity, a random stratified sampling scheme was applied whereby patients were classified according to age using the following categories: 18–59, 60–79, 80+. However, the youngest patient was 22 years of age. The SARS-CoV-2 infection was laboratory-confirmed by quantitative reverse transcription PCR (qRT-PCR) using FDA-approved CDC SARS-CoV-2 panel two [45]. Patients were randomly selected from within each group for a target of 95–97 per group. This resulted in 287 unique individuals during the period of March – May 2020. While demographic and clinical information were successfully collected from all 287 patient samples, leftover nasopharyngeal swab samples were available from 218 patients. The leftover nasopharyngeal swab samples for these 218 patients were used to evaluate immune and metabolic mediators at the site of infection–the nasal mucosa. Viral loads were determined in the samples from 174 patients.

### Multiplex cytokine, adipokines assays, and qRT-PCR for viral loads

**FIGURE 1:**
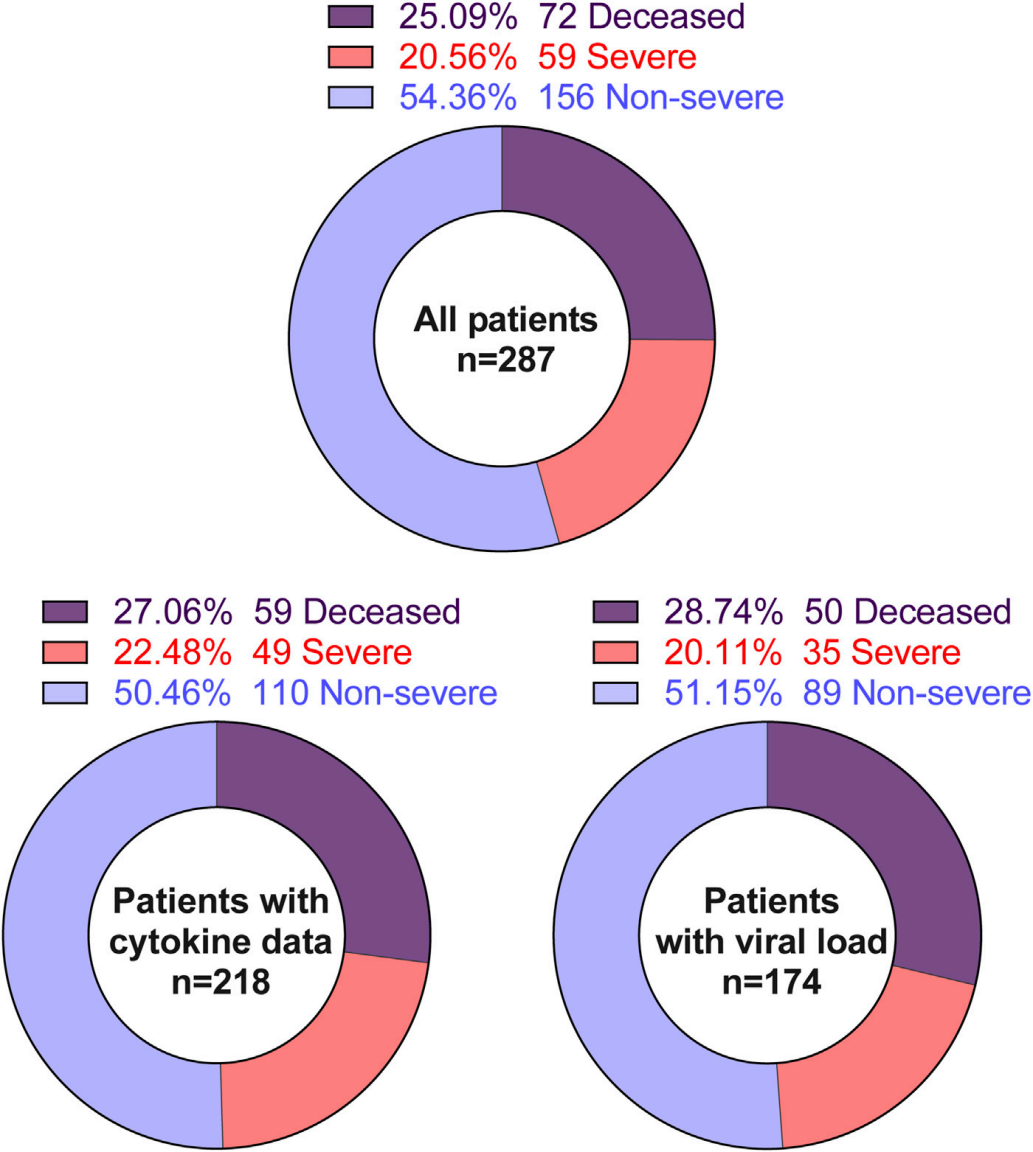
Summary of the studied population.

Nasopharyngeal swab samples were not available from all patients in our cohorts. Our available data are detailed in Figure 1. Cell-free supernatants from nasopharyngeal swab samples were incubated at 56°C for 20 min to deactivate SARS-CoV-2 virions [48]. Then, heat-treated supernatants were subjected to electrochemiluminescence-based multiplex assays according to the manufacturer’s protocol (MSD). The following analytes were measured: C-peptide, GLP-1, Glucagon, Insulin, IP-10, Leptin, PYY, G-CSF, GM-CSF, IFN-α2a, IFN-γ, IFN-β, IL-10, IL-12p70, IL-13, IL-15, IL-17A, IL-18, IL-1β, IL-1α, IL-22, IL-23, IL-29, IL-33, TSLP, IL-4, IL-5, IL-6, MCP-1, MCP-2, MCP-3, MCP-4, MDC, MIP-1α, MIP-1β, MIP-3α, MIP-3β, and MIP-5. Samples with analytes concentrations below the limit of detection (LOD) of the assay are replaced by a value equal to the LOD divided by the square root of 2 [49]. The data summaries are shown in Supplementary Table S1.

### Quantitative reverse transcriptase PCR for viral loads

Viral RNA nasopharyngeal swab samples were extracted, transcribed, and amplified as previously described [50]. The results were interpolated from an internal standard curve, produced by identical processing of serial dilution of a known copies-number of SARS-CoV-2 RNA stock (EDX, #COV019), hereafter referred to as the viral load.

### Data collection and coding

To investigate risk factors, anonymized clinical, paraclinical, and demographic data from patients admitted to Our Lady of the Lake (OLOL), were extracted into a REDCap database. To anonymize information from patients, the remnant nasopharyngeal swab samples from OLOL were relabeled with lab sample’s ID before being sent to our lab. The lab’s sample ID was used to communicate between OLOL and our lab.

Patient disposition was defined as “severe” if the patient was admitted to the intensive care unit (ICU) or “deceased” if the patient died due to COVID-19 during the hospitalization and within 30 days of discharge. All other patients were coded as “less severe.” Obesity was determined by body mass index calculated from weight and height in the medical record, based on the CDC adult definition of “obese” [51]. Biological sex, age, and race were obtained from the medical record. Race was collected as African American (AA), Caucasian, Asian, and Other.

Patient cardiac, renal, pulmonary, hepatic, vascular, cancer, diabetes, and connective tissue comorbidities, as well as age at infection were also collected. To evaluate the combined impact of these comorbidities on COVID-19 severity, we used weighted Charlson Comorbidity Index (CCI) [52], a sum of weighted scores for each comorbidity.

### Statistics

The data are described using standard descriptive statistics. The relationship of demographic, clinical, virological, and immunological variables with the outcome of disease severity (non-severe, severe, and deceased) was examined using ordered logistic regression. The effect of variables on the development of severe and deceased COVID-19 was represented as an unadjusted odds ratio (ORs) with 95% confidence interval (CI). Severity was tested for association with age, viral load, immune mediators, demographical variables, and obesity status with ordinal logistic regression while multinomial and bivariate logistic regression were employed to test for association between viral load and race and sex, respectively. Correlation matrixes for analytes was conducted using non-parametric Spearman correlation with two-tailed p-values and 95% confidence interval (95% CI).

We use an exploratory approach to ensure a final model for predicting COVID-19 severity. The initial multivariable ordered logistic regression model included only variables with P ≤ 0.05 from the bivariate analyses. Because continuous variables were collected in different metrics, we used standard deviation units to standardize all variables [53]. Details regarding the selected variables are provided in Supplementary Table S1. A final ordered logistic regression model retained only variables with P ≤0.05.

Then, we used the final ordered logistic regression model to calculate the coefficients of a formula to predict a logit transformation of the probability of severe and critical COVID-19, respectively. Thus, the logarithm of the odds is log [P(Y ≤ j)/P(Y > j)] = logit [P(Y ≤ j)] = α_j_ - ∑β_i_X_i_ [54]. With Y is an ordinal outcome with J the degree of disease severity (j = 1 for non-severe, j = 2 for severe, and j = 3 for deceased), P (Y ≤ j) is the cumulative probability of Y less than or equal to a specific degree of disease severity. X is the value of the particular predictors included in the final model. There are i = 3 predictors in our parsimonious model (i = 1, 2, and 3). α_j_ is the intercept for specific (j) degree of disease severity; β_i_ is the vector of regression coefficient or the effect of the individual (i) predictors on the specific outcome Y – degree of disease severity.

Statistical significance was assessed at the **α** = 0.05 level. The associations were evaluated using odds ratios (OR) with 95% confidence interval (95% CI) and *p ≤ 0.05, **p ≤ 0.01, ***p ≤ 0.01, and ****p ≤ 0.0001 as statistically significant. All statistics were performed in R (version 4.0.4) in R Studio (1.4.17) and SPSS (IBM).

## Results

### Characterization of the study cohort

**FIGURE 2:**
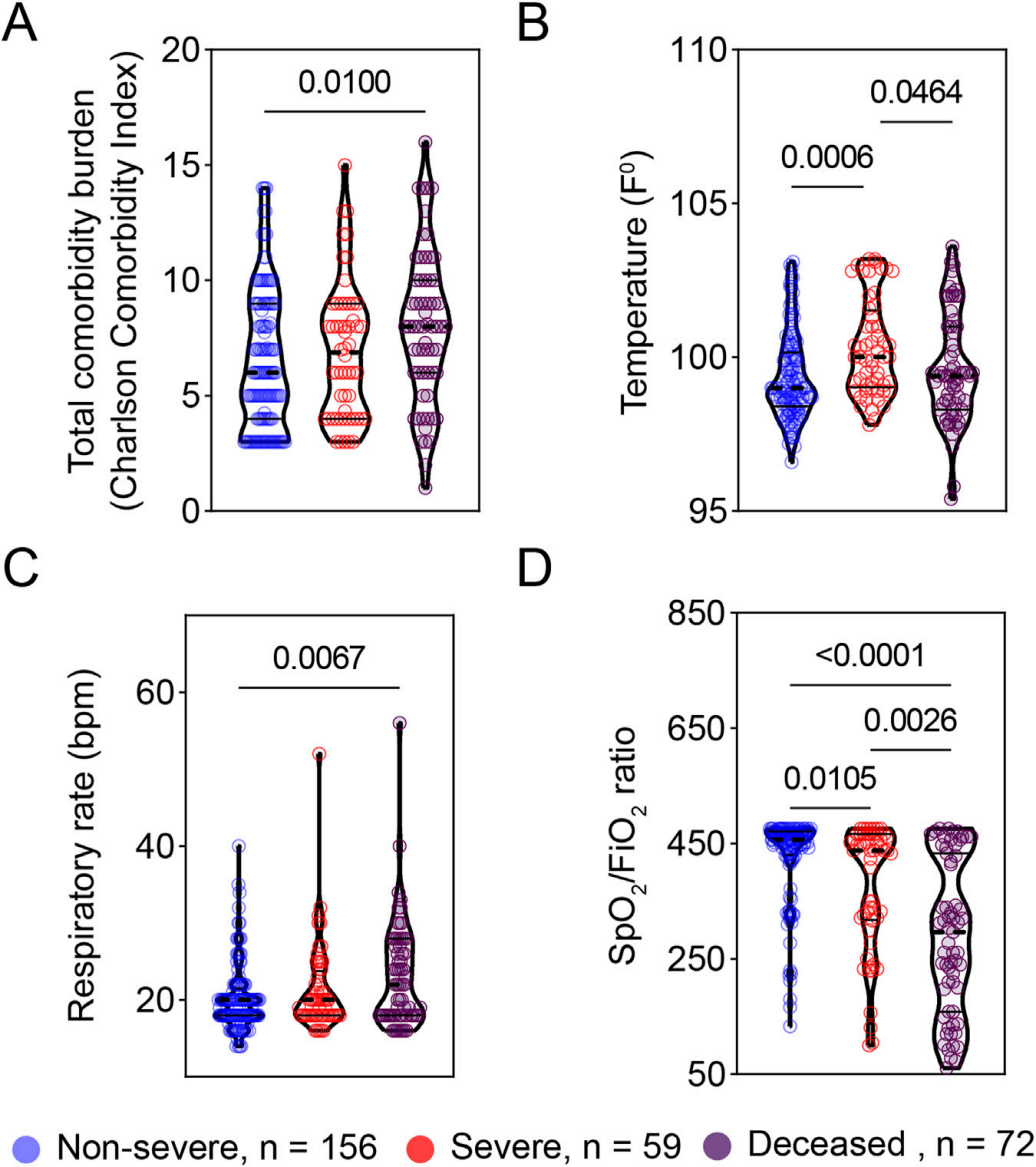
Vital signs at triage are associated with COVID-19 severity. The early vital signs at triage were compared between COVID-19 patients with non-severe (n = 156) and severe symptoms (n = 59) and deceased patients (n = 72) and are represented as violin plots. **(A)** Total comorbidity burden; **(B)** Temperature; **(C)** Respiratory rate; and **(D)** SpO_2_/FiO_2_ rate. The median is represented by the middle line. Significance was determined using Kruskal-Wallis non-parametric with post-hoc Dunn’s multiple comparison test. P ≤ 0.05 is considered as significant. ns: non-significant.

We included 287 COVID-19 patient samples in our study. These patients were admitted to local hospitals in Baton Rouge from March 2020 to May 2020 with a range of COVID-19 severity, including 72 deceased, 59 severe, and 156 non-severe patients (Table 1). Patients with severe COVID-19 were hospitalized for significantly more days compared to patients with less severe COVID-19 (Table 1). While race and ethnicity are not significantly different between the three groups, age and gender were not uniformly distributed across the three groups (Table 1). Although body mass index (BMI) as a continuous variable is not associated with COVID-19 severity, the BMI-based obesity classification is significantly different among the three groups (Table 1). We also evaluated the relationship of more than 20 significant comorbidities with COVID-19 severity (Supplementary Table S1). Among these comorbidities, the preexisting conditions related to hepatic, renal, cardiac, and pulmonary diseases were significantly associated with COVID-19 severity (Table 1). To evaluate the total burden of these comorbidities on COVID-19 severity, we used the Charlson Comorbidity Index (CCI) (53). Accordingly, the clinical severity significantly worsened as CCI increased (Figure 2B).

**TABLE 1:** Demographic and clinical characteristics of enrolled COVID-19 patients.

Characteristics	Deceased (n = 72)	Severe (n = 59)	Non_severe (n = 156)	P-value
Age (Median [IQR])	70.00 [58.75, 82.50]	64.00 [54.50, 74.00]	61.00 [48.00, 73.25]	0.002
LOS (Median [IQR])	8.00 [4.00, 12.25]	7.00 [4.00, 13.50]	3.00 [1.00, 5.00]	<0.001
ICU LOS (Median [IQR])	2.00 [0.00, 6.00]	3.00 [2.00, 7.50]	0.00 [0.00, 0.00]	<0.001
BMI (Median [IQR])	28.82 [25.91, 37.42]	32.22 [27.86, 39.70]	31.06 [25.03, 37.19]	0.346
Temperature at triage (Median [IQR])	99.25 [98.30, 100.38]	99.95 [99.03, 101.22]	99.00 [98.35, 100.30]	0.004
Heart rate at triage (Median [IQR])	99.00 [89.00, 110.00]	98.00 [86.00, 111.25]	93.00 [80.00, 106.00]	0.112
Respiration rate at triage (Median [IQR])	22.00 [18.00, 28.00]	20.00 [18.00, 23.25]	19.50 [18.00, 21.25]	0.004
SBP at triage (Median [IQR])	125.00 [106.00, 135.50]	122.50 [110.00, 140.25]	127.50 [115.00, 141.00]	0.15
DBP at triage (Median [IQR])	71.00 [63.00, 83.00]	72.00 [63.00, 83.50]	75.00 [66.00, 83.25]	0.381
SpO2 at triage (Median [IQR])	95.00 [91.00, 97.00]	96.00 [93.00, 98.00]	97.00 [94.00, 99.00]	0.003
Sex (%)				<0.001
Female	25 (34.7)	36 (61.0)	106 (67.9)	
Male	47 (65.3)	23 (39.0)	50 (32.1)	
Race (%)				0.386
African American	51 (70.8)	45 (76.3)	100 (64.1)	
Caucasian	17 (23.6)	13 (22.0)	49 (31.4)	
Others	4 (5.6)	1 (1.7)	7 (4.5)	
Ethnicity (%)				0.581
Hispanic	0 (0.0)1	(1.7)2	(1.3)	
Non_hispanic	72 (100)	58 (98.3)	154 (98.7)	
Obesity (%)* (missing 10 data points)				<0.001
Healthy	14 (19.7)	6 (10.2)	48 (32.7)	
Obesity	33 (46.5)	33 (55.9)	67 (45.6)	
Overweight	24 (33.8)	17 (28.8)	29 (19.7)	
Underweight	0 (0.0)	3 (5.1)	3 (2.0)	
Significant comorbidities present				
Chronic pulmonary disease (%)	9 (12.5)	4/55 (6.8/93.2)	2/154 (1.3/98.7)	0.049
Mild liver disease (%)	2/70 (2.8/97.2)	0/59 (0.0/100.0)	0/156 (0.0/100.0)	0.049
Peripheral vascular disease (%)	3/69 (4.2/95.8)	1/58 (1.7/98.3)	0/156 (0.0/100.0)	0.043
Acute kidney injury = Checked/Unchecked (%)	22/50 (30.6/69.4)	11/48 (18.6/81.4)	25/131 (16.0/84.0)	0.038
Congestive Heart Failure = No/Yes (%)	50/16 (75.8/24.2)	47/5 (90.4/9.6)	126/18 (87.5/12.5)	0.042
COPD = No/Yes (%)	55/11 (83.3/16.7)	47/5 (90.4/9.6)	137/7 (95.1/4.9)	0.019
Liver Disease (%)				0.008
Mild	2 (3.0)	1 (0.7)	3 (5.8)	
Moderate to Severe	3 (4.5)	0 (0.0)	0 (0.0)	
None	61 (92.4)	143 (99.3)	49 (94.2)	
Diabetes Mellitus (%)				0.003
End-organ damage	19 (28.8)	13 (25.0)	25 (17.4)	
None or diet-controlled	27 (40.9)	25 (48.1)	98 (68.1)	
Uncomplicated	20 (30.3)	14 (26.9)	21 (14.6)	

Definition of abbreviation: IQR, interquartile range; LOS, length of stay; ICU, intensive care unit; BMI, body mass index; SBP and DBP, systolic and diastolic blood pressure; SPO2, oxygen saturation; COPD, chronic obstructive pulmonary disease.

### The early vital signs at triage are associated with COVID-19 severity

To explore early predictors of disease progression, we next examined the relationship of vital signs at triage with disease severity. At triage, patients with severe COVID-19 or deceased COVID-19 patients exhibited significantly higher body temperature and respiratory rate as compared to patients with non-severe COVID-19 (Figures 2B, C). Also, oxygen saturation to fraction of inspired oxygen ratios (SpO_2_/FiO_2_) at triage were significantly greater in non-severe COVID-19 patients compared to their severe or deceased peers (Figure 2D). There was no significant difference in either systolic (SBP) or diastolic blood pressure (DBP) among the three groups studied (Table 1).

### Local mucosal inflammatory responses and respiratory viral load associated with COVID-19 severity

**FIGURE 3:**
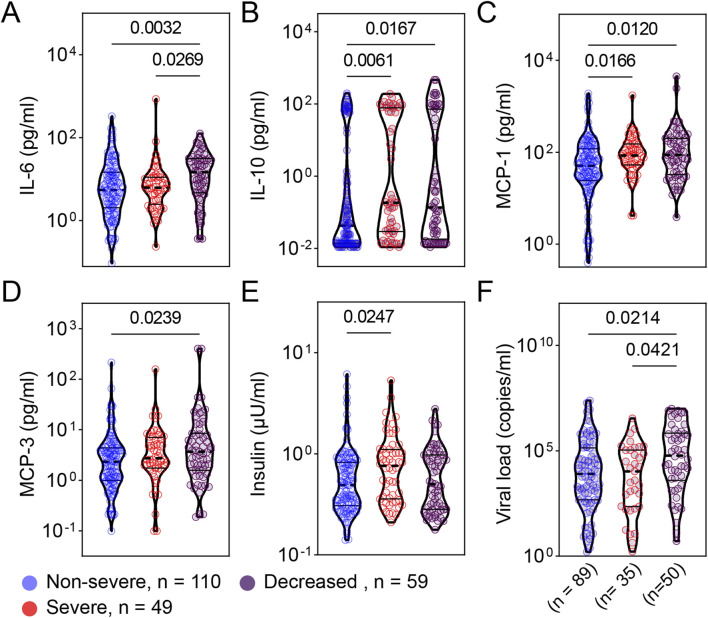
Patients with more severe COVID-19 exhibited greater levels of respiratory cytokines, adipokines, and viral load. Cytokines [IL-6 **(A)**, IL-10 **(B)**], chemokines [MCP-1 **(C)**, MCP-3 **(D)**], adipokines [insulin **(E)**], and viral load **(F)** from samples collected at admission were compared between patients with non-severe (n = 110), severe (n = 49), and deceased (n = 59) COVID-19. **(A)** Total comorbidity burden; **(B)** Temperature; **(C)** Respiratory rate; and **(D)** SpO_2_/FiO_2_ rate. Viral load data were available from 89, 35, and 50 patients from the non-severe, severe, and critical COVID-19 groups, respectively. The comparison are illustrated as violin plots. The median is represented by the middle dashed line. Significance was determined using Kruskal-Wallis non-parametric with post-hoc Dunn’s multiple comparison test. P≤0.05 is considered as significant. ns: non-significant.

To evaluate the local mucosal inflammatory responses, we subjected remnant nasopharyngeal swab samples to electrochemiluminescence -based multiplex assays examining 7 adipokines and 38 cytokines and chemokines. These analytes cover a wide range of inflammatory and metabolic pathways. We found increased levels of respiratory IL-6 and IL-10 in patients with more severe COVID-19 compared to less severe patients (Figures 3A, B). Similarly, patients with severe and critical COVID-19 exhibited significantly higher levels of monocyte chemoattractant protein −1 and −3 (MCP-1 and MCP-3) (Figures 3C, D). Although respiratory levels of insulin were significantly higher in severe compared to non-severe patients, there was no difference between severe and deceased patients regarding insulin levels (Figure 3E). We found that the distributions of viral load were significantly different between non-severe vs. deceased and severe vs. deceased (Figure 3F).

### SARS-CoV-2 load significantly correlates with respiratory IL-10, MCP-1, and MCP-3

**FIGURE 4:**
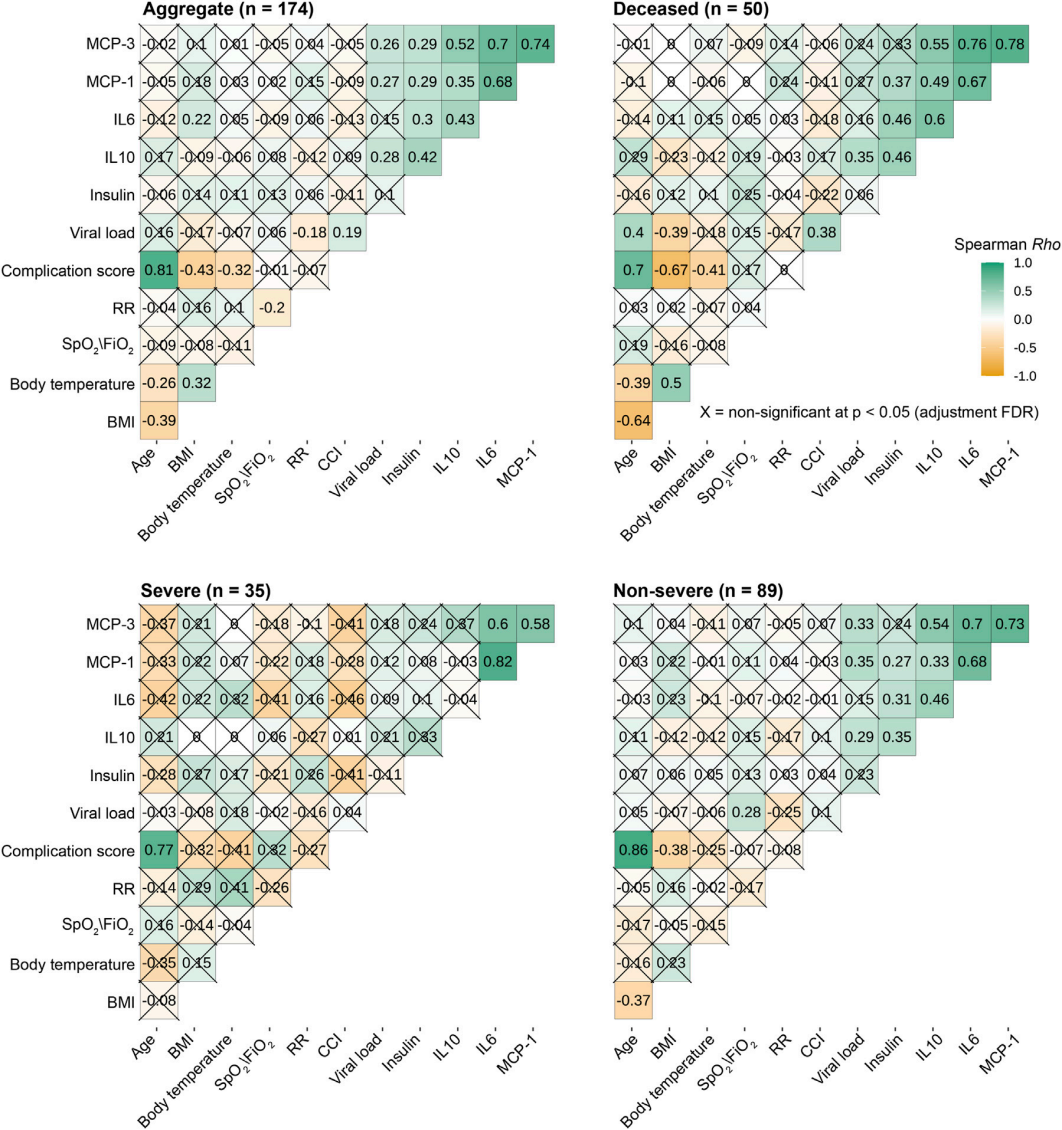
SARS-CoV-2 load significantly correlates with respiratory IL10, MCP-1 and MCP-3. The heat maps illustrate the Spearman rank correlation coefficient (Rho), varying from positive to negative correlation; green-white-dark yellow). The multiple comparison problems were controlled by adjusted False-discovery rate-adjusted P values with significant: P ≤ 0.05 (uncrossed squares) and non-significant P> 0.05 (crossed squares). BMI: body mass index; RR: respiratory rate; CCI: CCI: Total comorbidity burden – Charlson Comorbidity Index.

We next examined the relationship between viral load, local mucosal immune mediators, and early clinical indicators. To avoid the multiple comparisons problem, we only included variables significantly different between disease severity groups (Figure 4). We found that viral load was differentially correlated with immune response markers depending on whether data were aggregated over all patients or stratified based on disease severity (Figure 4). The pattern of correlation was not homogenous. In general, viral load was significantly and positively correlated with MCP-1, MCP-3, and IL-10. When we stratified for the degree of disease severity, several analytes were associated with viral load in one but not the other groupings. For example, IL-10 was positively and significantly correlated with viral load in deceased or non-severe patients but not in severe patients. Of note, there is no significant correlation between viral load and all other variables (Figure 4). Indeed, when we binned individuals into categories, ordered logistic regression revealed no association between increasing age group and viral load [OR: 0.86, 95% CI: (.73, 1.01)] (Supplementary Figure S1).

Next, we investigated whether viral load in this population was associated with being African American (“AA”) vs. not African American “(non-AA”). Logistic regression showed no association between viral load and identification as AA vs. not AA (Supplementary Figure S1). Similarly, we tested for an effect of biological sex (male vs. female) and found none (Supplementary Figure S1). On the other hand, viral load was significantly and positively correlated with age and CCI in deceased patients (Figure 4).

We observed a significant positive correlation among MCP-1, MCP-3, and IL-6 regardless of the degree of disease severity (Figure 4), suggesting common immune responses to SARS-CoV-2 infection. Although IL-10 and IL-6 were not correlated within the group of patients with severe COVID-19, they were directly proportional in deceased and non-severe groups.

Among early clinical and demographic indicators, we consistently observed a strong positive correlation between age at infection and CCI in all studied groups. In contrast, respiratory rate and SpO_2_/FiO_2_ ratio were not correlated with other variables regardless of disease severity. Intriguingly, in general, body temperature at triage was significantly correlated with BMI (Figure 4).

### Male individuals with reduced SpO2/FiO2 ratio and increased CCI are at greater risk of developing severe and critical COVID-19

**FIGURE 5:**
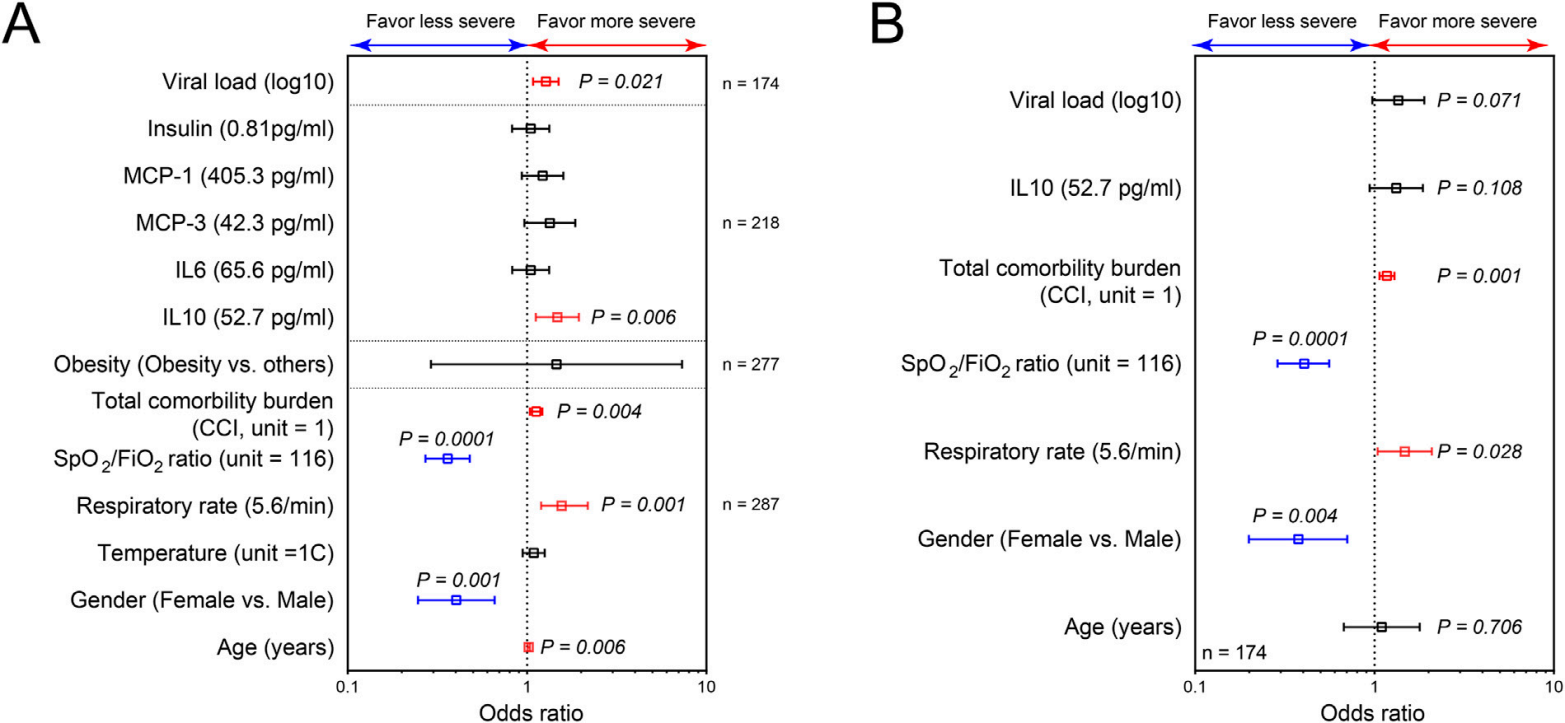
SARS-CoV-2 load, respiratory IL10, SPO_2_/FiO_2_ ratio, respiratory rate, gender and CCI at admission are significant risk factors for more severe COVID-19. Plots of risk factors for the development of more severe and critical COVID-19, using ordered logistic regression of individual factors **(A)**; and the combining effects for these potential risk factors **(B)**. The vertical line represents an odds ratio of 1. Odds ratios were represented as median with 95% confidence interval. P ≤ 0.05 is considered as significant.

Having demonstrated the association between clinical and immunological factors with COVID-19 severity, we next sought to evaluate their potential predictability for disease severity. First, the relationship of individual factors with the severity of disease was assessed using ordered logistic regression (Figure 5A). To establish parsimonious models, we only tested the relationship of the severity of disease with factors that are not uniformly distributed among studied groups (non-severe, severe and deceased groups). We found that female (OR, 0.35; 95% CI, 0.19–0.64) individuals with high SpO_2_/FiO_2_ ratio at triage (OR, 0.37; 95% CI, 0.27–0.51) are at 2.86- and 2.7 fold lower risk of developing severe/critical COVID-19, respectively (Figure 5A). In contrast, higher CCI, age at infection, viral load, respiratory IL-10, and respiratory rate at triage were the drivers of worse clinical outcome. There was no significant association of other variables with COVID-19 severity by ordered logistic regression (Figure 5A). However, it is noteworthy that BMI-based obesity was significantly different among the three groups of patients (Non-severed vs. Severe vs. Deceased) with greater numbers of overweight and obese individuals classified as severe (33 obese, 17 overweight, 6 healthy weight) or deceased (33 obese, 24 overweight, 14 healthy weight) as compared to healthy weight and this is reflected in Table 1.

**FIGURE 6:**
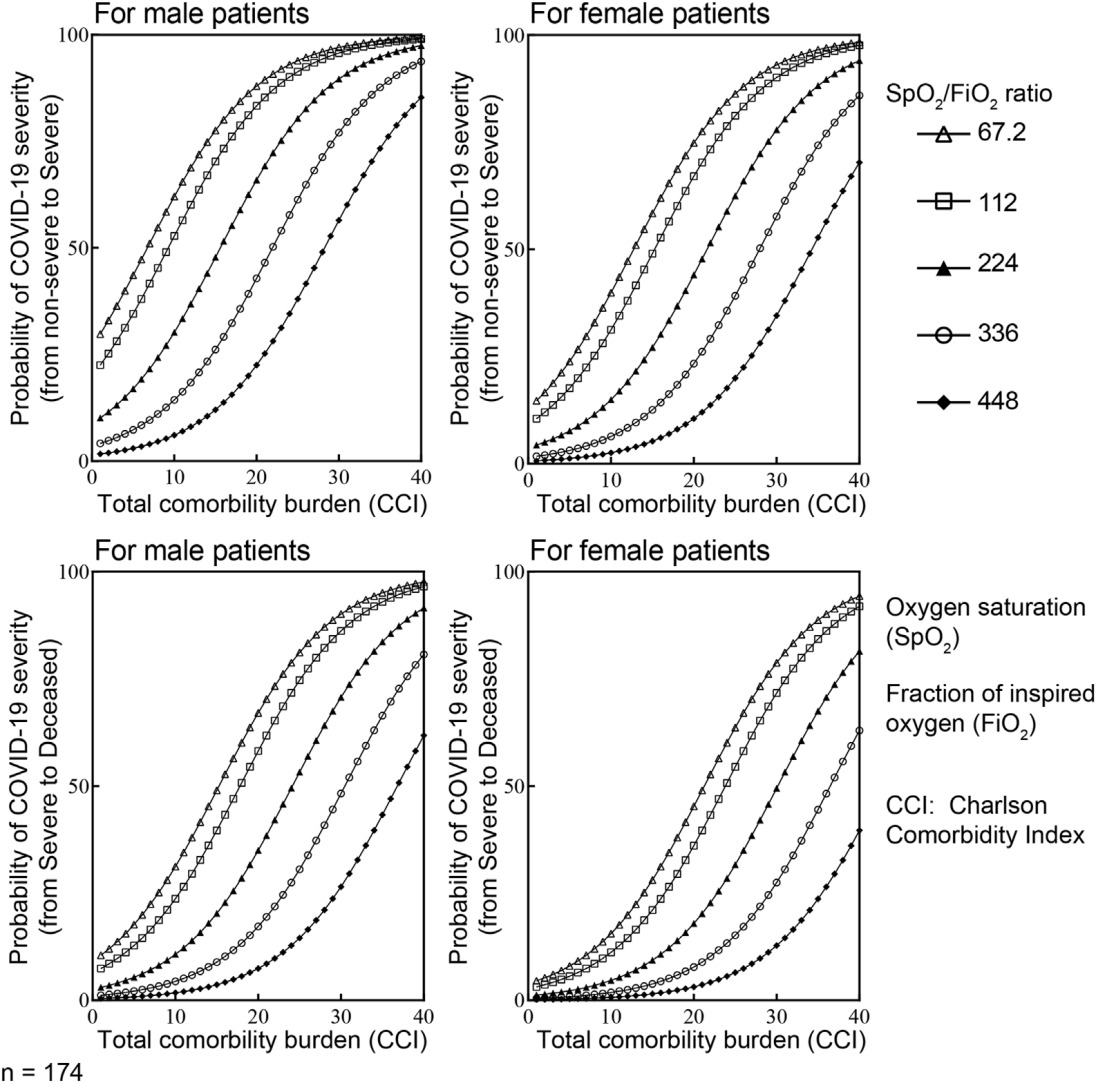
The combined influence of CCI, SpO_2_/FiO_2_ ratio at triage and gender on the development of more severe COVID-19 using ordered logistic regression. Considering Y is an ordinal outcome with J the degree of disease severity (j = 1 for non-severe, j = 2 for severe and j = 3 for deceased), P (Y ≤ j) is the cumulative probability of Y less than or equal to specific degree of disease severity. Therefore, the odds of being less than or equal to specific degree of disease severity is P(Y ≤ j)/P(Y > j). Accordingly, the logarithm of the odds is log [P(Y ≤ j)/P(Y > j)] = logit [P(Y ≤ j)]. With X is value of the particular predictors included in the final model. There are i = 3 predictors in our parsimonious model (i = 1, 2,3). α_j_ is the intercepts for specific (j) degree of disease severity; β_i_ is the vector of regression coefficient or the effect of individual (i) predictors on the specific outcome Y–degree of disease severity. In this context, logit [P(Y ≤ j)] = α_j_ - ∑β_i_X_i_. In the final ordered logistic regression model: α (non-severe vs. severe) = 1.34; α (severe vs. deceased) = 2.62; β_(male)_ = 0.901; β_(CCI)_ = 0.15; β_(SPO2/FiO2)_ = −0.95.

Among these 7 variables (gender, SpO_2_/FiO_2_ ratio at triage, CCI, respiratory IL-10, viral load, respiratory rate at triage, and age at infection), only CCI, SpO_2_/FiO_2_ ratio at triage, gender, and respiratory rate at triage were retained as potential risk factors in the final ordered logistic regression (Figure 5B). Because respiratory rate and SpO_2_/FiO_2_ ratio are clinically similar variables, only SpO_2_/FiO_2_ ratio, CCI, and gender were included in developing the parsimonious model. The combined influence of gender, SpO_2_/FiO_2_ ratio at triage, and CCI in the final ordered logistic regression model is illustrated in Figure 6. With the same levels of three risk factors, the probability of patients with non-severe COVID-19 developing severe disease (non-severe that escalates to severe) is higher than the probability of patients with severe COVID-19 progressing to more critical COVID-19 (severe that escalates to death). The effect of CCI and SpO_2_/FiO_2_ ratio at triage on the development of severe and deceased COVID-19 was greater in patients identifying as male. For example, a female patient with SPO_2_/FiO_2_ ratio of 224 at triage and CCI of 10 has a 14.9% probability of developing severe COVID-19 (if admitted without severe symptoms) and a 4.6% probability of progressing to critical/deceased COVID-19 (if admitted with severe symptoms). These probabilities will be significantly higher for male patients. For a male patient with the same SpO_2_/FiO_2_ ratio of 224 at triage and CCI of 10, the probabilities increase to 30.2% (non-severe progressing to severe) and 10.7% (severe progressing to critical/deceased). Therefore, males with reduced SpO_2_/FiO_2_ ratio and increased CCIs are at greater risk of developing severe and critical/deceased COVID-19.

## Discussion

Our current study demonstrated that SpO_2_/FiO_2_ ratio at triage, CCI, and gender are significant risk factors for developing severe and critical COVID-19 (Figure 5B). By leveraging these early indicators, the study established a practical prognostic tool for COVID-19 severity (Figure 6). As the variables included in our model are readily available and easily collected upon admission, this tool could prove valuable for COVID-19 management in the clinical setting and possibly other acute respiratory distress syndrome (ARDS) scenarios.

Using the residual nasopharyngeal swab samples, our study demonstrates the significant association between respiratory immune mediators (including IL-10, IL-6, MCP-1, and MCP-3) and COVID-19 severity (Figures 3A–D). In addition, our data reveal that viral load at the time of admission is associated with disease severity or mortality among hospitalized patients during the first wave of SARS-CoV-2 in Louisiana, USA (Figure 3F). This contributes to the growing evidence that viral load is a potential indicator of COVID-19 severity and a prognostic marker [10–19].

Numerous studies have proposed prediction models for COVID-19 severity [55–59]. In these previous studies, immune mediators in peripheral blood were used to predict the outcome of COVID-19. Although these immune mediators are strongly associated with COVID-19 severity and were shown as independent indicators for the progression of COVID-19, they were detected during the late acute phase of the disease (day 5–20 post-symptoms onset) and more likely resulted from developing severe disease [55–59]. Moreover, these immune mediators are not always easily accessible in clinical settings or available in a timely manner. These limitations hinder the predictability of immune-related variables in forecasting COVID-19 severity. In contrast, our study employs early and common clinical indicators, including SpO_2_/FiO_2_ ratio at triage, CCI, and gender, to create straightforward and convenient prognostic model for COVID-19 severity (Figure 6).

Considering the complexity and dynamic nature of COVID-19 progression, which rapidly changes throughout the disease course, we designed our model to calculate the probability of non-severe patients developing severe COVID-19 and the probability of severe patients developing more critical/deceased symptoms separately. As a result, our model offers a supplementary tool for assessing the risk of developing severe and critical/deceased COVID-19 in clinical settings at the time of admission without the need for additional paraclinical parameters. Additionally, we provide a method for designing parsimonious prognostic models for other viral respiratory infection diseases (Figure 5). However, due to the moderate sample size (n = 174), it is necessary to validate the predictive capacity of our proposed model in larger, independent cohorts.

Our current study also examined immunological mediators and viral load from remnant nasopharyngeal swab samples. It is evident that the exacerbated pro-inflammatory responses to SARS-CoV-2 are significantly associated with more severe COVID-19 immunopathology [41–47]. While studies have provided insights from immune mediator levels in peripheral blood following SARS-CoV-2 infection, the immune responses in the peripheral blood compartment may not accurately reflect the local immune responses at a more relevant infected site. Herein, we demonstrated the significant association between elevated levels of respiratory immune mediators (including IL-10, IL-6, MCP-1, and MCP-3) and COVID-19 severity. MCP-1 and MCP-3, produced mainly by cells such as airway epithelial cells, endothelial cells, and myeloid cells, were found to be monocyte chemotactic proteins for myeloid and lymphoid cells [60]. The increased MCP-1 and MCP-3 in more severe COVID-19 reinforce the pathogenic role of the exacerbated pro-inflammatory responses in COVID-19.

Using remnant nasopharyngeal swab samples, which are more accessible and less invasive than peripheral blood collection, we not only reinforce the previous findings on the association of increased circulating immune mediators and more severe COVID-19 [41–47, 61, 62] but more importantly demonstrate the feasibility of evaluating the humoral component of local mucosal immune responses and viral load at the infection site. This method allows us to evaluate the viral load and mucosal immune responses at the site of SARS-CoV-2 infection at the same time point, helping understand the relationship between viral infection and mucosal immune responses at the early of infection. Increased MCP-1 has been shown to be significantly and negatively correlated with the inhibition of interferon regulatory factor 3 (IRF3) pathway in patients with severe COVID-19 [63]. It is evident that the SARS-CoV-2 spike and Nsp12/6 proteins attenuate the host’s innate immune responses by suppressing IRF3-induced type 1 IFN production [64–66], suggesting the mechanism of immune evasion to facilitate the viral replication and COVID-19 severity. Together these data suggest that increased MCP-1 levels are significantly and negatively correlated with attenuated type 1 IFN production and, therefore, positively correlated with elevated viral load. Indeed, we found that increased viral load is associated with more severe COVID-19 and significantly, positively correlated with IL-10, MCP-1, and MCP-3 (Figures 3F, 4). Our data confirm the previous studies on the positive association of excessive cytokines and increased viral load with COVID-19 severity.

However, unlike data on peripheral cytokine responses, findings on viral load in association with COVID-19 severity are highly inconsistent among studies [18, 22] due to several factors. First, the disparities in the timing of sample collection contribute to inconsistency, as viral replication kinetics rapidly change over the course of the disease due to host immune responses. Controlling for varying degrees of different stages of the disease is essential for interpreting viral load data. However, these confounding effects were often not examined in numerous studies [10–27]. When available, the peak of viral load in longitudinal samples can be used to standardize the findings across studies. Second, viral load data were determined in different specimens, including respiratory [21] and plasma samples [67]. It has been shown that viremia is associated with severe and critical COVID-19 and is often under the limit of detection in asymptomatic and less severe patients [68]. The viremia in severe and critical COVID-19 patients is not comparable with viral load in respiratory samples, which is available in patients with a full spectrum of the disease severity (from asymptomatic to critical).

Our current study has certain limitations. During the first wave of COVID-19 (from March to May 2020), there was an extremely urgent need to identify the SARS-CoV-2 infected patients among patients with respiratory-related illnesses using nasopharyngeal swabs. Early in the pandemic, most COVID-19 patients at our institution were in isolation, making it difficult to collect longitudinal specimens and all relevant patient information, such as the day of symptom onset. We are aware that the single-time point respiratory sample collection in our study only represents a snapshot of changes in local immune responses. Because viral replication and immune responses to viral infection change throughout the disease, it is clear that the temporal profiles of infection need to be considered to get a clear picture of the relationship between viral load and immune responses. Nevertheless, our data demonstrate that even using remnant nasopharyngeal swab samples collected at admission and stored frozen for several months, we are able to observe early local mucosal responses to SARS-CoV-2 at the infected site. Once again, our study emphasizes the importance of considering the relationship between viral load and immune responses as investigating the mechanism of COVID-19 pathology. It also shows the value of early detection of risk factors and potential prognostic markers for clinical management. Nonetheless, the study also notes the need for validation in larger cohorts.

## Data Availability

The original contributions presented in the study are included in the article/Supplementary Material, further inquiries can be directed to the corresponding authors.
