# Supplementary material for: Predicting severe COVID-19 using readily available admission indicators: SpO2/FiO2 ratio, comorbidity index, and gender
Source: Exp Biol Med (Maywood). 2024 Nov 20;249:10193. doi: 10.3389/ebm.2024.10193 (PMC11614601; doi:10.3389/ebm.2024.10193)

SUPPLEMENTARY MATERIALS

Table S1. Descriptive Statistics of respiratory cytokine, adipokines, and viral load in our cohorts.

(pg/ml)	All patients					Deceased patients					Severe patients					Non-severe patients				
	N	Minimum	Maximum	Mean	Std. Deviation	N	Minimum	Maximum	Mean	Std. Deviation	N	Minimum	Maximum	Mean	Std. Deviation	N	Minimum	Maximum	Mean	Std. Deviation
C-peptide	218	8.829	334.839	119.774	64.600	59	8.829	237.753	119.038	57.023	49	16.454	279.616	131.386	63.064	110	8.829	334.839	114.997	68.865
GLP-1	218	0.311	14.601	6.695	3.173	59	0.570	12.624	6.433	3.627	49	0.311	14.601	6.927	3.087	110	1.160	13.781	6.732	2.964
Glucagon	218	0.034	26.237	13.450	5.884	59	0.037	26.237	13.070	6.769	49	0.609	23.528	13.643	5.472	110	0.034	25.978	13.568	5.590
**Insulin**	218	0.140	6.116	0.754	0.801	59	0.177	2.768	0.686	0.549	49	0.210	5.313	0.944	0.941	110	0.140	6.116	0.706	0.840
IP-10	218	0.129	13688.713	5646.862	3445.887	59	1.056	13688.713	5465.444	3719.638	49	231.712	12963.652	6309.928	3476.405	110	0.129	12448.538	5448.803	3271.111
Leptin	218	0.196	2282.751	130.286	267.453	59	3.122	1487.623	137.816	271.320	49	0.196	1084.929	122.207	190.174	110	2.797	2282.751	129.845	295.378
**MCP-1**	218	0.258	4468.786	163.781	405.252	59	0.258	4468.786	232.460	648.473	49	4.176	1731.502	142.402	243.961	110	0.510	1901.366	136.468	271.052
PYY	218	0.465	22.983	4.867	3.159	59	0.465	9.784	4.884	2.449	49	0.506	19.173	5.413	3.968	110	0.465	22.983	4.614	3.089
G-CSF	218	0.179	12571.712	687.071	1577.154	59	0.190	12571.712	950.833	2094.845	49	2.913	3609.335	648.345	1020.034	110	0.179	9945.864	562.849	1450.799
GM-CSF	218	0.022	2.057	0.162	0.219	59	0.022	2.057	0.193	0.340	49	0.022	0.752	0.172	0.163	110	0.022	0.762	0.141	0.146
IFNα2α	218	0.091	728.368	37.890	93.334	59	0.091	728.368	56.413	135.662	49	0.091	466.015	44.164	94.432	110	0.091	266.404	25.161	57.057
IFNβ	218	0.420	258.681	24.783	39.533	59	0.420	207.782	25.551	43.814	49	1.494	130.168	20.641	30.877	110	0.420	258.681	26.216	40.743
IFNγ	218	0.042	517.732	16.089	54.319	59	0.160	389.125	22.223	68.883	49	0.042	165.906	13.635	31.667	110	0.054	517.732	13.891	53.663
**IL-10**	218	0.000	467.963	21.961	52.749	59	0.011	467.963	32.212	76.031	49	0.000	192.060	25.826	47.220	110	0.000	199.259	14.741	36.949
IL-12p70	218	0.002	217.056	14.168	37.316	59	0.002	207.432	18.787	48.445	49	0.021	190.466	15.539	34.029	110	0.006	217.056	11.080	31.488
IL-13	218	0.000	4.196	0.625	0.480	59	0.187	4.196	0.698	0.595	49	0.000	3.771	0.672	0.534	110	0.001	3.579	0.566	0.370
IL-15	218	0.034	114.513	14.572	25.571	59	0.034	114.513	19.325	32.729	49	0.699	80.924	15.863	24.311	110	0.043	108.532	11.447	21.182
IL-17A	218	0.019	54.601	2.174	6.006	59	0.057	48.616	2.101	6.585	49	0.048	15.393	1.606	2.742	110	0.019	54.601	2.467	6.722
IL-18	218	0.477	9248.090	1148.138	1758.658	59	0.477	9248.090	1295.234	2029.858	49	7.903	5384.707	1253.391	1645.810	110	1.820	7608.010	1022.356	1655.125
IL-1α	218	0.182	1321.498	127.355	206.609	59	0.182	1069.800	173.938	277.149	49	7.128	1321.498	150.943	234.592	110	0.517	623.516	91.861	131.071
IL-1β	218	0.005	165.575	4.765	15.964	59	0.010	106.818	6.985	18.980	49	0.005	165.575	6.002	23.768	110	0.019	68.556	3.022	7.775
IL-22	218	0.050	85.029	4.153	10.917	59	0.050	76.914	6.600	15.420	49	0.050	85.029	5.146	12.977	110	0.050	29.660	2.398	5.459
IL-23	218	0.632	542.408	4.257	36.701	59	0.632	542.408	11.281	70.455	49	0.632	5.898	1.851	1.493	110	0.632	10.481	1.561	1.609
IL-29	218	0.495	153.255	15.433	23.569	59	0.495	153.255	16.948	28.543	49	0.595	126.675	19.407	27.293	110	0.578	76.669	12.849	18.145
IL-33	218	0.087	4683.431	220.135	633.887	59	0.131	1915.727	170.010	407.578	49	0.087	2462.181	265.734	576.445	110	0.110	4683.431	226.709	750.252
IL-4	218	0.009	128.426	8.220	16.776	59	0.009	128.426	10.878	22.692	49	0.009	41.832	9.690	15.433	110	0.009	53.448	6.139	13.124
IL-5	218	0.002	89.343	3.355	9.229	59	0.002	89.343	4.465	12.797	49	0.007	40.167	4.194	8.469	110	0.009	37.441	2.385	6.994
**IL-6**	218	0.093	857.530	21.355	65.620	59	0.363	125.361	21.371	24.875	49	0.233	857.530	27.298	121.769	110	0.093	326.742	18.700	41.117
MCP-2	218	0.043	1833.272	46.192	178.939	59	0.048	1833.272	81.693	285.415	49	0.045	364.148	26.516	54.037	110	0.043	1374.843	35.914	134.720
**MCP-3**	218	0.099	402.408	10.400	42.302	59	0.189	402.408	19.867	72.644	49	0.099	157.213	7.814	22.340	110	0.099	214.072	6.475	21.626
MCP-4	218	6.607	445.065	60.997	95.994	59	9.571	352.240	71.410	106.299	49	8.862	445.065	73.608	106.755	110	6.607	347.826	49.795	84.006
MDC	218	11.347	12786.618	420.282	980.267	59	11.347	12786.618	603.635	1704.423	49	22.101	2119.897	471.733	598.114	110	11.347	1814.993	299.019	418.878
MIF	218	66.370	35760.188	28954.411	3594.379	59	66.370	35336.074	28183.906	4702.144	49	21291.323	35760.188	29449.309	3043.332	110	15245.077	35580.172	29147.228	3067.239
MIP-1α	218	2.641	2988.662	164.462	305.849	59	7.431	2988.662	262.732	500.437	49	3.164	791.363	153.981	192.451	110	2.641	1040.116	116.422	170.451
MIP-1β	218	0.278	2248.559	124.332	234.294	59	0.278	2248.559	188.589	391.567	49	2.895	374.374	103.732	107.196	110	0.645	675.511	99.044	139.880
MIP-3α	218	1.338	10521.373	310.339	852.354	59	1.338	2128.794	270.613	459.936	49	4.120	10521.373	516.830	1563.687	110	1.338	3328.164	239.664	481.638
MIP-3β	218	0.291	6373.490	661.230	710.664	59	0.413	2836.346	632.338	649.760	49	29.006	2245.947	741.544	604.589	110	0.291	6373.490	640.950	784.902
MIP-5	218	0.023	3506.543	804.370	589.231	59	0.023	2874.722	850.347	652.879	49	90.469	3506.543	941.055	640.797	110	3.135	2749.249	718.822	516.370
TSLP	218	0.044	36.139	2.646	4.402	59	0.048	22.285	2.908	4.456	49	0.044	25.264	3.153	5.105	110	0.044	36.139	2.280	4.031
Viral load (log10)*	174	0.190	7.380	4.023	1.686	50	0.710	6.990	4.620	1.633	35	0.210	6.540	3.665	1.651	89	0.190	7.380	3.828	1.661

*Viral load is represented as log10 of absolute number of viral RNA copies. Variables in bold and underlined were included in the initital multivariable ordered logistic regression model.

Figure S1. The association of viral load and age, body mass index (BMI), sex and race.

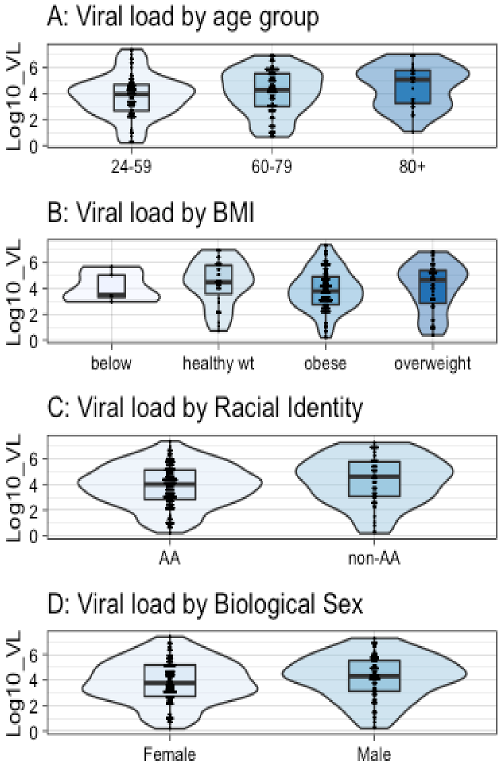

Supplement: Supplementary file 1 [file DataSheet1.PDF]
